# Connecting the Dots: Exploring the Interplay Between Preeclampsia and Peripartum Cardiomyopathy

**DOI:** 10.1155/2024/7713590

**Published:** 2024-06-25

**Authors:** Khanisyah Erza Gumilar, Khairunnisa binti Abd Rauf, Muhammad Ilham Aldika Akbar, Nareswari Cininta Imanadha, Susetyo Atmojo, Alisia Yuana Putri, Erry Gumilar Dachlan, Gus Dekker

**Affiliations:** ^1^ Department of Obstetrics and Gynecology Faculty of Medicine Universitas Airlangga, Surabaya, Indonesia; ^2^ Department of Obstetrics and Gynecology Hospital of Universitas Airlangga, Surabaya, Indonesia; ^3^ Department of Obstetrics and Gynecology Dr Soetomo General Hospital, Surabaya, Indonesia; ^4^ National Cardiovascular Center Harapan Kita, Jakarta, Indonesia; ^5^ Department of Cardiology Faculty of Medicine Universitas Airlangga, Surabaya, Indonesia; ^6^ Women's and Children's Division Lyell McEwin Hospital Medical School North University of Adelaide, Adelaide, Australia

**Keywords:** endothelial dysfunctions, genetic, inflammatory, peripartum cardiomyopathy, preeclampsia

## Abstract

Preeclampsia and peripartum cardiomyopathy (PPCM) are significant obstetric problems that can arise during or after pregnancy. Both are known to be causes of maternal mortality and morbidity. Several recent studies have suggested a link between preeclampsia and the pathophysiology of PPCM. However, the common thread that connects the two has yet to be thoroughly and fully articulated. Here, we investigate the complex dynamics of preeclampsia and PPCM in this review. Our analysis focuses mainly on inflammatory and immunological responses, endothelial dysfunction as a shared pathway, and potential genetic predisposition to both diseases. To begin, we will look at how excessive inflammatory and immunological responses can lead to clinical symptoms of both illnesses, emphasizing the role of proinflammatory cytokines and immune cells in modifying vascular and tissue responses. Second, we consider endothelial dysfunction to be a crucial point at which endothelial damage and activation contribute to pathogenesis through increased vascular permeability, vascular dysfunction, and thrombus formation. Finally, we examine recent information suggesting genetic predispositions to preeclampsia and PPCM, such as genetic variants in genes involved in the management of blood pressure, the inflammatory response, and heart structural integrity. With this synergistic study, we seek to encourage more research and creative therapy solutions by emphasizing the need for an interdisciplinary approach to understanding and managing the connection between preeclampsia and PPCM.

## 1. Introduction

Preeclampsia and peripartum cardiomyopathy (PPCM) are two different health issues that can occur during or after pregnancy. Preeclampsia involves high blood pressure and can harm organs like the kidneys. PPCM is a rare type of heart failure that happens in the last month of pregnancy or within 5 months after giving birth.

Recent studies reveal that these conditions might be related because they both involve problems with blood vessels, stress on cells, and inflammation [1–3]. However, the explanation for this requires further investigation. This is an important necessity because the clinical diagnosis and treatment for the two illnesses are significantly different. Another obstacle to understanding the relationship between these two conditions is that some aspects have yet to be thoroughly explored.

Here, we will discuss three factors that may correlate the two at an upstream level. We identify at least three common threads between preeclampsia and PPCM. Inflammatory and immunological responses, endothelial dysfunction, and genetic predisposition all play essential roles in the development of preeclampsia and PPCM.

This research has the potential to fundamentally alter the treatment of preeclampsia and PPCM patients. More research is needed to address the difficulties raised by both and provide fresh insights into how to treat preeclampsia and PPCM in the clinical setting.

### 1.1. A Synopsis of Preeclampsia

Preeclampsia is a potentially dangerous pregnancy illness characterized by high blood pressure and organ damage, with the kidney and liver being the most affected organs [4, 5]. Preeclampsia affects over 4 million women globally each year, resulting in the deaths of over 70,000 women and 500,000 newborns. It usually happens after the 20th week of pregnancy and can cause several complications for both the mother and the fetus [6]. Although placental problems are often considered to be the primary cause, preeclampsia is a complex disease and has been attributed to a wide variety of etiologies, including genetic [7], immunological [8, 9], and vascular [10] factors.

Preeclampsia is gestational hypertension accompanied by one or more of the following new-onset conditions at ≥ 20 weeks of gestation: proteinuria and other maternal end-organ dysfunctions, including neurological complications, lung edema, hematological problems, kidney and liver injuries, and uteroplacental dysfunction [11]. On the other hand, preeclampsia can affect numerous organ systems and cause a wide range of symptoms such as generalized swelling, stomach pain, nausea, vomiting, rapid weight gain, headaches, shortness of breath, and vision issues. Preeclampsia can progress to eclampsia, a serious condition characterized by convulsions that, if left untreated, can endanger both mother and fetal health [12].

**Figure 1:**
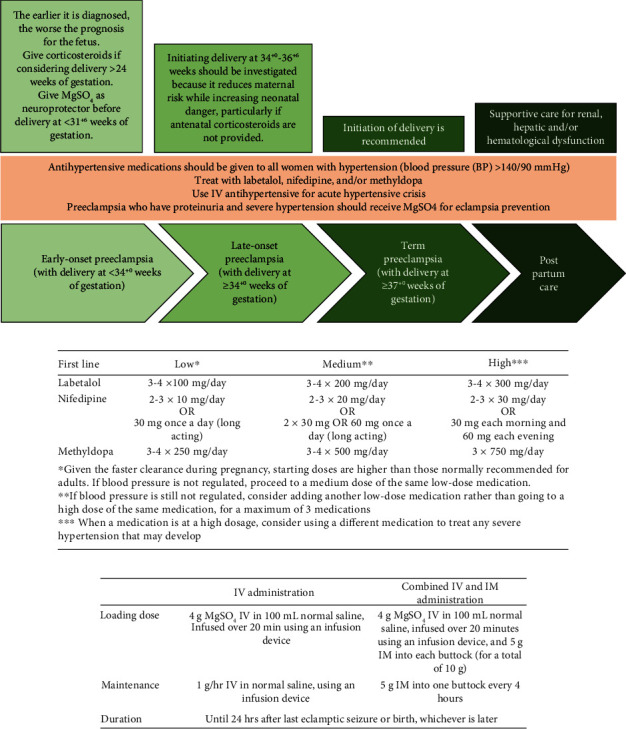
(a) Management algorithm for preeclampsia. (b) Antihypertensive therapy for nonurgent control of hypertension in pregnancy (modified from Magee et al.) [11]. (c) MgSO_4_ protocol in preeclampsia (modified from Brown et al.) [13].

Prenatal care is essential for detecting preeclampsia early, including blood pressure monitoring and urine testing. Antihypertensive agents, magnesium sulfate as an anticonvulsant [13], hospitalization, and, in severe cases, early delivery of primarily premature newborns to avert future complications are all options for management. Women with a history of preeclampsia or other risk factors may be closely monitored during their pregnancies [14]. Because of the extent and complexity of preeclampsia, gestational age can be used as a basis for determining the management of this problem (Figure 1). Doppler velocimetry for uterine artery screening is another step toward early identification of preeclampsia. Similarly, when paired with other screening approaches, biomarkers like as soluble FMS-like tyrosine kinase 1 (sFlt-1) and placental growth factor (PlGF) can improve the accuracy of preeclampsia identification [12, 14].

Briefly, preeclampsia is a complex and possibly hazardous syndrome that necessitates attentive prenatal care and prompt action. Understanding the symptoms and risk factors is critical for healthcare providers to give effective therapy and ensure the best outcomes for both mother and infant.

### 1.2. PPCM: An Overview

PPCM is a relatively rare idiopathic and potentially fatal illness that affects women in their late pregnancy or the early months after childbirth. It is distinguished by the development of heart failure, a condition in which the heart is unable to properly pump blood to meet the body's needs, and objective evidence of left ventricular systolic dysfunction. PPCM, unlike other types of cardiomyopathies, presents primarily around the time of delivery. According to NHLBI [15] and ESC [16], PPCM can be defined using a set of agreed-upon criteria (Table 1).

PPCM prevalence varies significantly across countries, regions, and ethnic groups. Nigeria had the highest frequency of 1 in 102 deliveries, while Japan had the lowest incidence of 1 in about 15,000 births. According to US studies, the occurrence rate of PPCM is highest among African-Americans, ranging from 1 in 439 to 1 in 421 deliveries. PPCM is a substantial contributor to maternal mortality and has been connected to an increase in maternal mortality rates [17].

The precise cause of PPCM is unknown; however, it is thought to be a combination of genetic susceptibility [18], hormonal [19], and environmental factors [20]. One theory is that the increased load on the cardiovascular system during pregnancy, combined with hormonal changes, may contribute to heart muscle weakness in vulnerable people [21].

The majority of PPCM occurrences occur after childbirth, usually during the first week. However, a small number of cases have been reported throughout the second and third trimesters of pregnancy [21]. Clinical presentations of congestive heart failure, such as shortness of breath, tiredness, orthopnea, paroxysmal nocturnal dyspnea, limb edema, and an enlarged heart, are all symptoms of PPCM [22]. Symptoms could be similar to those of a normal pregnancy or other myocardial problems, resulting in a delayed or missed diagnosis and affecting outcomes. Early diagnosis is crucial for optimum management, and doctors may use a variety of diagnostic procedures to assess heart function, including echocardiography [23, 24] and cardiac magnetic resonance [25, 26]. Echocardiography has the capability to reveal various cardiac issues, such as left ventricular systolic dysfunction, left ventricular dilatation, right ventricular enlargement, biatrial enlargement, four-chamber enlargement, and mitral and tricuspid regurgitation, along with elevated pulmonary pressure.

The latest treatment method for PPCM is called the BOARD (Bromocriptine, Oral heart failure therapy, Anticoagulation, vasoRelaxing agents, and Diuretics) approach [27]. This approach uses a team of specialists to address different aspects of the condition all at once, aiming to reduce symptoms and help improve the long-term health of patients. It is important that treatment is customized for each patient and decided upon by a team that includes experts in cardiology and obstetrics.

Bromocriptine is used to inhibit prolactin release from the pituitary gland. Specifically, the 16-kDa prolactin fragment has angiostatic, proapoptotic, and proinflammatory effects that can damage blood vessels, restricting oxygen and nutrient supply to the heart and potentially leading to heart failure. Bromocriptine is aimed at mitigating this damage and accelerating heart function recovery. For managing heart failure, oral medications reduce fluid retention, beta-blockers lessen the heart's workload and improve symptoms, and ACE inhibitors or ARBs enhance heart function recovery; these latter drugs are used postdelivery due to their teratogenic risks during pregnancy. Anticoagulants are employed to prevent thromboembolic complications, especially critical in PPCM patients with slowed blood flow or left ventricular dysfunction. Vasorelaxing agents are advised for those with systolic blood pressure above 110 mmHg. Additionally, diuretics help manage the fluid overload commonly associated with heart failure [27, 28].

Cases of PPCM that arise during late pregnancy, though rarer than after childbirth, need careful management. Certain drugs like ACE inhibitors and treatments that significantly reduce body fluid levels should be avoided. If a patient with heart issues is stable, a vaginal delivery is generally preferred unless there is a strong reason for a cesarean section. It is critical to monitor the patient's heart function closely during delivery. Epidural analgesia is typically favored for pain management. However, if a woman with severe heart failure remains unstable despite treatment, an immediate delivery may be necessary, no matter how far along the pregnancy is. In such situations, a cesarean section using central neuraxial anesthesia is recommended. Epidural anesthesia is chosen to prevent sudden changes in pressure or volume, but it needs to be precisely controlled by an experienced anesthesia team [28, 29].

### 1.3. Understanding the Link Between Preeclampsia and PPCM Is Critical

Recognizing the complex link between preeclampsia and PPCM is critical in maternal health. Preeclampsia, a prenatal hypertension health problem, might act as an initiator or exacerbating factor for PPCM, an uncommon but potentially fatal condition in which the heart fails before or after childbirth. Several things were found that can connect the two as problems that need attention (Table 2). Understanding this link is essential for healthcare providers who want to provide complete and proactive care to pregnant women.

Preeclampsia and hypertension are significantly linked to PPCM. A meta-analysis revealed that the total prevalence of preeclampsia was more than four times the 3% to 5% population prevalence [35]. It is important to note that preeclampsia can cause pulmonary edema despite the absence of PPCM. Even without clinical heart failure, certain echocardiographic studies have demonstrated that preeclampsia results in diastolic dysfunction [39–41]. Preeclampsia causes considerable cardiac injury, which can be clinically undetectable but can potentially manifest as pulmonary edema with maintained EF or as part of PPCM. However, it is also vital to recognize that PPCM is more than just a symptom of severe preeclampsia.

Our periodic case investigations in tertiary hospitals have also revealed the high prevalence of preeclampsia in PPCM patients. The prevalence of preeclampsia diagnosis surpassed 80% of all PPCM patients [42–45]. In addition, studies of eclampsia in the US show PPCM as a complication in the peripartum period. With an odds ratio (OR) of 12.88, PPCM was the more common complication, exceeding amniotic embolism (OR 11.94) and venous thromboembolism (OR 10.71) [46].

A study also found that PPCM with concurrent preeclampsia was linked with higher morbidity and mortality, as well as distinct patterns of LV remodeling and recovery of LV function as compared to PPCM without preeclampsia [47]. One critical issue raised is that PPCM is more than just a symptom of severe preeclampsia. Although uncommon, localized myocardial fibrosis has been also reported in this study [48]. Meanwhile, a meta-analysis study mentioned that PPCM and HPD-PPCM share different clinical profiles as well as types of remodeling, which may affect each disease's response to pharmacological treatment [49]. Eventually, there is a considerable connection and overlap between the two, implying that their pathophysiological mechanisms may be comparable [50]. As a result, even in the presence of preeclampsia, suspicion of PPCM should not be lowered, as this may impede timely treatment. It is a challenge for clinicians to differentiate between preeclampsia with PPCM or without it.

## 2. Shared Pathophysiological Features

### 2.1. Inflammatory and Immune Responses

Both preeclampsia and PPCM have important inflammatory and immunological responses, providing insight into the complex nature of both pregnancy-related disorders. Preeclampsia is characterized by systemic inflammation and immune system activation, both of which lead to vascular endothelial dysfunction [8, 9, 51]. Proinflammatory cytokines and immune cell activation cause preeclampsia and organ damage. While the exact origins of PPCM are unknown, it is known that an enhanced immune response and inflammation can contribute to cardiac muscle weakening, which contributes to the pathophysiology of PPCM [52, 53].

In preeclampsia and PPCM, several proinflammatory cytokines can be employed as markers (Table 3). Their roles in endothelial damage and cardiac muscle structure are said to be comparable. The proinflammatory cytokines in question can also be utilized as metrics to assess the patient's state in this scenario.

The presence of inflammatory and immunological responses in both illnesses suggests that they may share a pathogenesis. These responses could be linked, influencing the progression and severity of preeclampsia and PPCM. Recognizing similarities in inflammatory and immunological processes offers the door to targeted treatment approaches.

It is important to highlight that, while preeclampsia and PPCM share certain immunological and inflammatory traits, each illness is different. The complex interplay of these responses in the context of pregnancy-related illnesses underscores the need for additional study to elucidate the processes and find novel therapeutic targets that can successfully address both preeclampsia and PPCM.

Apart from the proinflammatory cytokines, the role of antiangiogenic factors plays an important role in the pathophysiology of preeclampsia and PPCM. Soluble sFlt-1 is an antiangiogenic protein secreted by the placenta antagonist to vascular endothelial growth factor (VEGF) and PlGF. sFlt-1 normally increases toward the end of pregnancy and declines rapidly after delivery [19]. In preeclampsia, there are increased circulating sFlt-1 associated with low level of free circulating VEGF and PlGF resulting in endothelial dysfunction [66]. In PPCM, level of sFlt-1 remains higher than normal after delivery. VEGF is also strongly antagonized in late gestation by the placental secretion of sFlt-1. A study has also shown that exogenous sFlt1 was sufficient to cause profound systolic dysfunction in mice lacking cardiac PGC-1*α*, even in the absence of pregnancy. PGC-1*α* is known to be proangiogenic and playing role in antioxidant regulation [67]. The high level of sFlt1 in women with preeclampsia may explain why preeclampsia is a strong risk factor and closely associated with PPCM.

### 2.2. Endothelial Dysfunction as a Common Pathway

Endothelial dysfunction, a condition characterized by increased vasoconstriction and alterations in hemostasis (platelet aggregation) and angiogenesis processes, has been identified as a central pathophysiological factor in both preeclampsia [10, 68] and PPCM [19]. These two conditions, although occurring in different clinical contexts, show characteristics of vascular inflammation, oxidative stress, and activation of coagulation pathways that disrupt normal endothelial function. Endothelial dysfunction causes hypertension, proteinuria, and an increased inflammatory response in preeclampsia. This is mostly due to imbalanced angiogenic factor release and endothelial cell destruction, which causes vasoconstriction and placental malfunction [69]. Meanwhile, PPCM is linked to cardiac endothelial dysfunction, which causes vascular remodeling and decreased nitric oxide availability, deteriorating myocardial function and eventually leading to heart failure [70]. According to an additional study, medicines aiming at restoring endothelial function may be a promising therapy strategy for both disorders [71].

**Figure 2:**
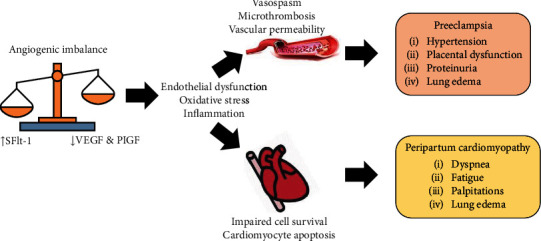
Shared pathophysiology between preeclampsia and PPCM.

Endothelial dysfunction is a result of an angiogenic imbalance caused by elevated SFlt-1 to VEGF and PlGF levels (Figure 2). Endothelial dysfunction, in conjunction with oxidative stress and inflammation, causes vasospasm, microthrombosis, and increased permeability, resulting in a variety of clinical symptoms in preeclampsia. Endothelial dysfunction, on the other hand, produces various phenomena in the heart muscle, including disturbance of cardiac muscle cell resilience and accelerated cell death. This will have a major impact when heart failure symptoms such as shortness of breath, tiredness, edema, and palpitations emerge in PPCM.

In addition, a study revealed a pathophysiological process involving unbalanced oxidative stress and increased prolactin cleavage into angiostatic and proapoptotic 16-kDa subfragments, which induces microRNA-146a (miR-146a) expression in endothelial cells, endothelial damage, and ventricular dysfunction [72]. The important role of endothelial damage is further supported by the significantly elevated endothelial microparticles found in acute PPCM, exhibiting apoptosis with impaired microcirculation.

Understanding endothelial dysfunction as a common cause of preeclampsia and PPCM opens the door to more targeted therapies. Endothelial function preservation or restoration therapies may be effective in slowing the advancement of certain disorders and lowering associated cardiovascular risks. This emphasizes the significance of a comprehensive approach to maternal health, identifying common pathophysiological pathways in preeclampsia and PPCM.

### 2.3. Potential Genetic Predispositions

Both preeclampsia and PPCM appear to be caused by genetic predispositions, highlighting the complex interplay between genetic variables and these pregnancy-related diseases [73]. Preeclampsia susceptibility appears to have a hereditary component, with a higher prevalence found in women with a family history of the disorder. Preeclampsia risk has been linked to genetic differences in genes involved in vascular function, immunological response, thrombophilia, and blood pressure regulation.

We attempted to trace the presence of genes related to PPCM using the database from https://www.ncbi.nlm.nih.gov/gene. A total of 13 genes associated with PPCM were discovered (Supporting Information S1). We searched for a connection between these genes and preeclampsia. Based on our investigation, we identified eight genes that have a role in preeclampsia and PPCM (Table 4).

Similarly, current research in the field of PPCM reveals a hereditary foundation for the development of this poor cardiac function. Investigations into familial clustering of PPCM in certain cases have suggested that a subset of PPCM may be a part of the spectrum of familial dilated cardiomyopathy (DCM), presenting in the peripartum period. PPCM may share genetic mutations like idiopathic DCM. The distribution of truncating variants in a large series of women with PPCM was found to be remarkably similar to those found in patients with idiopathic DCM. Two-thirds of identified truncating variants were in the titin gene (*TTN*), which encodes the sarcomere protein titin [103, 104]. Other variants in genes related to cardiomyocyte structure, cardiac remodeling, and angiogenesis are being investigated for their relevance in PPCM predisposition.

The common genetic basis of preeclampsia and PPCM suggests that overlapping genetic variables may influence both illnesses. In both conditions, specific genes with a major impact are unlikely to exist, since it is always the case in reproduction—this would have led to evolutionary gene “suicide.” However, understanding these genetic susceptibility tendencies is critical for risk prediction, early detection, and individualized management techniques. Genetic screening and counseling may become standard components of prenatal treatment to identify women at higher risk, allowing for targeted therapies and better monitoring to reduce the impact of these diseases on mother and fetal health. Further research into the complex genetic landscape of preeclampsia and PPCM has the potential to advance our understanding and lead to the development of more effective preventive and treatment measures.

## 3. Conclusion

Preeclampsia and PPCM are two significant pregnancy-related disorders that share pathophysiological aspects such as inflammatory and immunological responses, endothelial dysfunction, and probable genetic predispositions. These similarities point to a complex interaction of genetic, immunological, and vascular variables that contribute to the genesis and course of both illnesses. Understanding the complex link between preeclampsia and PPCM is essential for developing focused therapy options and improving maternal and fetal outcomes. Early detection and management of these illnesses are critical, and healthcare providers must maintain a high index of suspicion for these conditions, particularly in women with established risk factors. A multifaceted strategy that combines patient education, genetic counseling, and individualized therapy can help reduce the risks associated with these disorders. More study is needed to uncover the genetic foundations of preeclampsia and PPCM, as well as to discover novel therapies that can effectively address the multiple characteristics of the conditions.

## Figures and Tables

**Table 1:** Current definition of PPCM.

**NHLBI (2000)** • Heart failure in the last month of pregnancy or within 5 months of delivery • Absence of an identifiable cause for the cardiac failure • Absence of recognizable heart disease before the last month of pregnancy • LV systolic dysfunction identified by echocardiographic criteria, such as EF < 45% or fractional shortening less than 30%, or both
**ESC (2010)** • Heart failure secondary to LV systolic dysfunction with an EF < 45% • Occurrence toward the end of pregnancy or in the months following delivery (mostly in the months following delivery) • No other identifiable cause of heart failure
**Echocardiographic findings** • LVED dimension > 2.7 cm/m^2^ • M-mode fractional shortening < 30% • LVEF < 45%

Abbreviations: EF, ejection fraction; ESC, European Society of Cardiology; LV, left ventricle; LVED, left ventricular end-diastolic; NHLBI, National Heart, Lung, and Blood Institute.

**Table 2:** Association between preeclampsia and PPCM.

**Findings**	**Ref.**
Maternal age	[22, 30, 31]
Multiple pregnancies	[32–34]
Hypertension during pregnancy was significantly associated with PPCM	[3, 35–38]

**Table 3:** Proinflammatory cytokines in preeclampsia and PPCM.

	**Role in preeclampsia and PPCM**	**Ref.**
TNF-*α*	• Higher TNF-*α* levels were associated with lower mean LVEF • Degenerative changes and an increase in inflammation in decidua cells in preeclamptic placental villi • Affects the functioning of endothelial cells by increasing the vascular permeability and induces apoptosis of the trophoblastic cells	[54–58]

IL-1*β*	• The NLRP3 inflammasome generates ROS and has an impact on endothelial damage and systemic inflammation • IL-1*β* could induce PTX3 upregulation, which led to the inhibition of the proliferation, invasion, and cell cycle of trophoblasts • IL-1*β* causes atrophy, impairs contractility and relaxation, and decreases the deformation of cardiomyocytes	[59–62]

IL-6	IL-6 and its receptor trans-signaling represent an important cytokine axis in the pathogenesis of inflammation-associated disorders, including cardiovascular diseases, diabetes, and IUGR	[52, 63–65]

**Table 4:** Gene involvement in preeclampsia and PPCM.

	**Role in preeclampsia and PPCM**	**Ref.**
CD274 (PD-L1)	PD-L1 regulates the immune system and is linked to severe inflammation in preeclampsia. More PD-L1 can impair blood vessel function and damage the placenta. The precise relationship between PD-L1 and PPCM is uncertain. However, it may influence how the immune system responds and raises stress levels in the body during PPCM.	[74–77]

FLT1 (Fms-related tyrosine kinase 1)	FLT1 is a VEGF and PlGF receptor. In preeclampsia, elevated levels of sFlt1 have been identified, which bind VEGF and PlGF, limiting their bioavailability and inducing endothelial dysfunction. FLT1 may be involved in PPCM via angiogenesis and vascular homeostasis pathways. Disruptions in these pathways have been linked to cardiovascular dysfunction.	[78–80]

GNB3 (Guanine nucleotide-binding protein subunit beta-3)	GNB3 is a signaling transduction protein that has been linked to blood pressure regulation. GNB3 mutations or polymorphisms may raise the risk of preeclampsia. GNB3's relevance in PPCM is unknown, but given its role in cardiovascular function, changes in this gene may alter the risk or progression of PPCM.	[81–83]

MIR146A	miR-146a is a microRNA that regulates inflammatory responses as well as oxidative stress. miR-146a expression has been altered in preeclampsia, indicating a role in the disease's pathophysiology. miR-146a's effects on inflammatory responses and cellular homeostasis may play a role in PPCM. Changes in miR-146a expression or activity may contribute to cardiac dysfunction.	[84–87]

PDCD1 (PD-1)	PD-1 is an immune checkpoint that controls immune tolerance and protects against autoimmunity. In preeclampsia, disruptions in the PD-1 pathway may contribute to inappropriate immune responses and inflammation. PD-1, like PD-L1, may play a role in modifying immunological responses in PPCM. PD-1 expression or signaling dysregulation could influence the onset and severity of PPCM.	[88–90]

PlGF (placental growth factor)	PlGF levels are frequently reduced in preeclampsia, leading to placental and endothelial dysfunction. PlGF's role in PPCM is unknown, but abnormalities in angiogenesis and tissue perfusion associated with low PlGF levels may contribute to PPCM pathophysiology.	[91–94]

SERPINE1 (serpin family E member 1)	SERPINE1 encodes the PAI-1 inhibitor, which controls fibrinolysis. SERPINE1 expression is increased in preeclampsia, which contributes to thrombosis and vascular dysfunction. Although there is no direct link between SERPINE1 and PPCM, its role in coagulation and vascular homeostasis may impact pathological processes in heart diseases.	[95–99]

VEGFA (vascular endothelial growth factor A)	VEGFA is an important angiogenesis mediator. Endothelial dysfunction, hypertension, and proteinuria can all result from VEGFA pathway malfunction in pregnancy. VEGFA may contribute to the pathogenic mechanisms of PPCM, such as endothelial dysfunction and cardiac remodeling, through its role in angiogenesis and vascular integrity.	[100–102]

## Data Availability

We used databases from https://www.ncbi.nlm.nih.gov/gene to track the presence of genes associated with PPCM.

## Supporting Information

Supporting Information Additional supporting information can be found online in the Supporting Information section. S1 A total of 13 genes associated with peripartum cardiomyopathy can be downloaded at https://www.ncbi.nlm.nih.gov/gene.
