# Supplementary material for: Connecting the Dots: Exploring the Interplay Between Preeclampsia and Peripartum Cardiomyopathy
Source: J Pregnancy. 2024 Jun 25;2024:7713590. doi: 10.1155/2024/7713590 (PMC11219213; doi:10.1155/2024/7713590)
Supplement: Supporting Information — Additional supporting information can be found online in the Supporting Information section. S1 A total of 13 genes associated with peripartum cardiomyopathy can be downloaded at https://www.ncbi.nlm.nih.gov/gene. [file 7713590.f1.docx]

This data was downloaded from https://www.ncbi.nlm.nih.gov/gene on October, 27th 2023

1. ADRB1

Official Symbol: ADRB1 and Name: adrenoceptor beta 1 [Homo sapiens (human)]

Other Aliases: ADRB1R, B1AR, BETA1AR, FNSS2, RHR

Other Designations: beta-1 adrenergic receptor; adrenergic, beta-1-, receptor; beta-1 adrenoceptor; beta-1 adrenoreceptor

Chromosome: 10; Location: 10q25.3

Annotation: Chromosome 10 NC_000010.11 (114043866..114046904)

MIM: 109630

ID: 153

2. CD274

Official Symbol: CD274 and Name: CD274 molecule [Homo sapiens (human)]

Other Aliases: B7-H, B7H1, PD-L1, PDCD1L1, PDCD1LG1, PDL1, hPD-L1

Other Designations: programmed cell death 1 ligand 1; B7 homolog 1; CD274 antigen; PDCD1 ligand 1

Chromosome: 9; Location: 9p24.1

Annotation: Chromosome 9 NC_000009.12 (5450542..5470554)

MIM: 605402

ID: 29126

3. CHRM2

Official Symbol: CHRM2 and Name: cholinergic receptor muscarinic 2 [Homo sapiens (human)]

Other Aliases: HM2

Other Designations: muscarinic acetylcholine receptor M2; 7TM receptor; acetylcholine receptor, muscarinic 2; muscarinic M2 receptor

Chromosome: 7; Location: 7q33

Annotation: Chromosome 7 NC_000007.14 (136868652..137020213)

MIM: 118493

ID: 1129

4. FLT1

Official Symbol: FLT1 and Name: fms related receptor tyrosine kinase 1 [Homo sapiens (human)]

Other Aliases: FLT, FLT-1, VEGFR-1, VEGFR1

Other Designations: vascular endothelial growth factor receptor 1; fms related tyrosine kinase 1; fms-like tyrosine kinase 1; fms-related tyrosine kinase 1 (vascular endothelial growth factor/vascular permeability factor receptor); tyrosine-protein kinase FRT; tyrosine-protein kinase receptor FLT; vascular permeability factor receptor

Chromosome: 13; Location: 13q12.3

Annotation: Chromosome 13 NC_000013.11 (28300346..28495128, complement)

MIM: 165070

ID: 2321

5. GNB3

Official Symbol: GNB3 and Name: G protein subunit beta 3 [Homo sapiens (human)]

Other Aliases: CSNB1H, HG2D

Other Designations: guanine nucleotide-binding protein G(I)/G(S)/G(T) subunit beta-3; G protein, beta-3 subunit; GTP-binding regulatory protein beta-3 chain; guanine nucleotide binding protein (G protein), beta polypeptide 3; guanine nucleotide-binding protein G(I)/G(S)/G(T) beta subunit 3; heterotrimeric guanine nucleotide-binding protein 2D; hypertension associated protein; transducin beta chain 3

Chromosome: 12; Location: 12p13.31

Annotation: Chromosome 12 NC_000012.12 (6840925..6847393)

MIM: 139130

ID: 2784

6. HSPB6

Official Symbol: HSPB6 and Name: heat shock protein family B (small) member 6 [Homo sapiens (human)]

Other Aliases: HEL55, Hsp20, PPP1R91

Other Designations: heat shock protein beta-6; epididymis luminal protein 55; epididymis secretory sperm binding protein; heat shock 20 kDa-like protein p20; heat shock protein family B (small) member B6; heat shock protein, alpha-crystallin-related, B6; protein phosphatase 1, regulatory subunit 91

Chromosome: 19; Location: 19q13.12

Annotation: Chromosome 19 NC_000019.10 (35754566..35757029, complement)

MIM: 610695

ID: 126393

7. MIR146A

Official Symbol: MIR146A and Name: microRNA 146a [Homo sapiens (human)]

Other Aliases: MIRN146, MIRN146A, miR-146a, miRNA146A

Other Designations: hsa-mir-146; hsa-mir-146a

Chromosome: 5; Location: 5q33.3

Annotation: Chromosome 5 NC_000005.10 (160485352..160485450)

MIM: 610566

ID: 406938

8. PDCD1

Official Symbol: PDCD1 and Name: programmed cell death 1 [Homo sapiens (human)]

Other Aliases: CD279, PD-1, PD1, SLEB2, hPD-1, hPD-l, hSLE1

Other Designations: programmed cell death protein 1; programmed cell death 1 protein; protein PD-1; systemic lupus erythematosus susceptibility 2

Chromosome: 2; Location: 2q37.3

Annotation: Chromosome 2 NC_000002.12 (241849884..241858894, complement)

MIM: 600244

ID: 5133

9. PGF

Official Symbol: PGF and Name: placental growth factor [Homo sapiens (human)]

Other Aliases: D12S1900L, PIGF, PLGF, PlGF-2, SHGC-10760, PGF

Other Designations: placenta growth factor; placental growth factor, vascular endothelial growth factor-related protein

Chromosome: 14; Location: 14q24.3

Annotation: Chromosome 14 NC_000014.9 (74941830..74955764, complement)

MIM: 601121

ID: 5228

10. PRL

Official Symbol: PRL and Name: prolactin [Homo sapiens (human)]

Other Aliases: GHA1

Other Designations: prolactin; decidual prolactin; growth hormone A1

Chromosome: 6; Location: 6p22.3

Annotation: Chromosome 6 NC_000006.12 (22287246..22302835, complement)

MIM: 176760

ID: 5617

11. SERPINE1

Official Symbol: SERPINE1 and Name: serpin family E member 1 [Homo sapiens (human)]

Other Aliases: PAI, PAI-1, PAI1, PLANH1

Other Designations: plasminogen activator inhibitor 1; endothelial plasminogen activator inhibitor; serine (or cysteine) proteinase inhibitor, clade E (nexin, plasminogen activator inhibitor type 1), member 1; serpin E1; serpin peptidase inhibitor, clade E (nexin, plasminogen activator inhibitor type 1), member 1

Chromosome: 7; Location: 7q22.1

Annotation: Chromosome 7 NC_000007.14 (101127104..101139247)

MIM: 173360

ID: 5054

12. TTN

Official Symbol: TTN and Name: titin [Homo sapiens (human)]

Other Aliases: CMD1G, CMH9, CMPD4, CMYP5, EOMFC, HMERF, LGMD2J, LGMDR10, MYLK5, SALMY, TMD

Other Designations: titin; connectin; rhabdomyosarcoma antigen MU-RMS-40.14

Chromosome: 2; Location: 2q31.2

Annotation: Chromosome 2 NC_000002.12 (178525989..178807423, complement)

MIM: 188840

ID: 7273

13. VEGFA

Official Symbol: VEGFA and Name: vascular endothelial growth factor A [Homo sapiens (human)]

Other Aliases: L-VEGF, MVCD1, VEGF, VPF

Other Designations: vascular endothelial growth factor A, long form; vascular endothelial growth factor A121; vascular endothelial growth factor A165; vascular permeability factor

Chromosome: 6; Location: 6p21.1

Annotation: Chromosome 6 NC_000006.12 (43770211..43786487)

MIM: 192240

ID: 7422
